# AI-assisted screening for mild cognitive impairment using routine EHR data: a Gradient Boosting approach

**DOI:** 10.3389/fneur.2026.1718791

**Published:** 2026-02-17

**Authors:** Tao Ye, Jianghua Peng

**Affiliations:** 1 School of Medicine, Shaoxing University, Shaoxing, China; 2 Department of General Practice, Shaoxing People’s Hospital (The First Affiliated Hospital, Shaoxing University), Shaoxing, China

**Keywords:** calibration, decision curve analysis, electronic health records, machine learning, mild cognitive impairment, risk prediction

## Abstract

**Objective:**

To develop and internally validate a machine learning (ML) model that identifies older outpatients with MCI using routine electronic health record (EHR) data.

**Methods:**

We conducted a retrospective cross-sectional study of community outpatients aged ≥60 years in Zhejiang, China. Structured EHR predictors included demographics, comorbidities/medications, lifestyle, and visit patterns. The outcome was adjudicated MCI based on cognitive screening (MoCA plus supplemental tests). Supervised ML classifiers were compared using 10-fold cross-validation and an independent held-out test set; class imbalance was addressed with SMOTE. Performance was assessed by the area under the ROC curve (AUC) and by sensitivity, specificity, positive predictive value (PPV), negative predictive value (NPV), accuracy, and F1 score.

**Results:**

The test set included ~640 patients (≈20% MCI). Gradient Boosting performed best: cross-validation mean AUC 0.855 (SD 0.031) and accuracy 0.862 (SD 0.013); test AUC 0.850, accuracy 0.833, and F1 0.402. At the default threshold, sensitivity was 0.286 and specificity 0.967 (PPV 0.679; NPV 0.847). Prioritizing sensitivity (~0.82) lowered specificity (~0.64). At a high-sensitivity threshold of 0.159, the model achieved a sensitivity of 0.802 with a specificity of 0.751 (PPV 0.441; NPV 0.939). Important predictors included older age, female sex, lower education, smaller family size, and higher depression scores.

**Conclusion:**

An ML model using routine outpatient EHR can discriminate MCI in older adults (AUC ≈ 0.85), supporting potential for automated, low-cost screening in primary care. Using the predicted probabilities generated in this analysis, we assessed calibration and conducted a decision-curve analysis. While the model shows good discrimination and calibration, external validation is still required to confirm clinical utility and refine operating thresholds.

## Introduction

Population aging has increased the public health burden of mild cognitive impairment (MCI). Recent reviews estimate a global MCI prevalence of about 23.7% (95% CI 18.6–29.6) in older adults (1). In China, a large meta-analysis of 393,525 adults aged ≥60 years reported a prevalence of 19.6% (2). Primary care settings face practical barriers—limited consultation time, workflow constraints, and communication challenges—that lead to underdiagnosis of cognitive impairment (3–5). Consequently, leveraging low-cost, routinely collected outpatient EHR data to identify individuals at risk for MCI is an attractive and scalable strategy.

Artificial intelligence (AI) and machine learning (ML) offer feasible tools for this task. Recent work shows that ML models using demographics, chronic disease burden, lifestyle, care-seeking behaviors, and basic cognitive/functional measures can achieve useful discrimination for Alzheimer’s disease and related disorders (ADRD) or MCI from EHR data (6). Other studies demonstrate that, even without imaging, Gradient-Boosting models like XGBoost can predict 3-year conversion risk among cognitively healthy older adults using non-imaging features (7). However, important evidence gaps remain: external validation and context-specific thresholding in community populations are limited, and beyond discrimination, reporting of calibration and clinical utility (e.g., decision-curve analysis) is often insufficient.

To address these issues, we developed and internally evaluated an MCI screening model based on community outpatient data, reporting transparent methods aligned with the TRIPOD+AI guidance (8) and framing model evaluation with contemporary BMJ guidance on prediction modeling and clinical utility (9, 10). The model can produce individual predicted probabilities; In this manuscript, we report discrimination (ROC–AUC) and operating characteristics at prespecified thresholds (sensitivity, specificity, PPV, NPV), in addition to discrimination (ROC–AUC) and classification metrics, we also present calibration plots and decision-curve analysis to assess clinical utility. External validation remains a future step. Thus, this internal analysis should be viewed as an exploratory first step rather than definitive evidence of clinical utility. We outline these future plans, including sample-size considerations for validation and model updating, in the Discussion.

## Methods

### Study design and reporting

We performed a retrospective cross-sectional analysis using de-identified records from community outpatient clinics. The study protocol received institutional ethics approval. We followed the STROBE guideline for observational studies (11). For model development and internal validation, we adhered to TRIPOD+AI standards (8). We also assessed risk of bias and applicability using PROBAST (12). The number of participants and outcome events (see Results) was considered adequate relative to the number of predictors, and formal sample-size calculations for external validation and model updating will follow the Riley et al. framework. A checklist mapping each TRIPOD+AI, STROBE, and PROBAST item to this manuscript or its Supplement is provided in the Supplementary material (12).

### Data source, participants, and outcome

Data were extracted from community health service centers in Zhejiang Province, China, including adults aged ≥60 years with at least one outpatient encounter in the past year. Inclusion required age ≥60 years, ≥1 outpatient visit in the previous 12 months, and sufficient data to ascertain cognitive status. We excluded individuals with established dementia or moderate–severe cognitive impairment, irrecoverable missingness in key variables, or conditions invalidating cognitive assessment (e.g., acute severe illness). The primary outcome was mild cognitive impairment (MCI), adjudicated according to the 2018 Chinese guideline (subjective cognitive complaint/corroboration, objective impairment in ≥1 domain, preserved basic activities of daily living, and not meeting dementia criteria) (13).

### Predictors and preprocessing

Candidate predictors were drawn from structured EHR fields: demographics (age, sex, education), social context (living alone, social participation), chronic conditions and medications (e.g., hypertension, diabetes, cerebrovascular disease, depressive symptoms), lifestyle factors (smoking, alcohol, physical activity, sleep), and care-seeking behaviors (visit frequency, department specialties). Continuous variables were screened for outliers and standardized; categorical variables were harmonized with consistent coding. Missing data were imputed by multiple imputation with chained equations (MICE) (14). We repeated model fitting in each imputed dataset and pooled the results. Since MCI was the minority class, we managed class imbalance in the training data using synthetic minority oversampling (SMOTE) and/or class weighting, while leaving the held-out test set unchanged. The choice of sampling strategy and evaluation metrics was informed by a recent review on imbalanced medical datasets (15).

### Data split and model development

We split the data at the participant level into a training set (80%) and an independent held-out test set (20%), ensuring no individual appeared in both. We evaluated 10 supervised learning algorithms: regularized logistic regression, support vector machine, k-nearest neighbors, naïve Bayes, decision tree, random forest, Gradient Boosting (LightGBM), extreme Gradient Boosting (XGBoost), and a multilayer perceptron neural network. Most models were implemented with scikit-learn, and boosting models used their native libraries (16). Hyperparameters were tuned via grid or Bayesian search within 10-fold cross-validation. Regularization and early stopping were applied where available to prevent overfitting.

### Performance evaluation and operating thresholds

Discrimination was summarized by the area under the receiver-operating-characteristic curve (ROC–AUC). We also reported sensitivity, specificity, PPV, NPV, and confusion matrices at two operating points: (i) a default threshold (maximizing Youden’s J statistic) and (ii) a high-sensitivity threshold (to minimize missed cases in community screening). Where sample size allowed, we obtained two-sided 95% confidence intervals for AUC and classification metrics by bootstrap resampling. Calibration (calibration plots, Brier score, calibration intercept and slope) was assessed using the individual predicted probabilities exported from the final model; decision-curve analysis was conducted using standard net-benefit calculations in accordance with contemporary guidance (9, 10).

### Implementation and reproducibility

We report 10-fold CV performance as mean ± SD and present AUC, accuracy, and F1 on the held-out test set. For proportions (sensitivity, specificity, PPV, NPV), two-sided 95% CIs were computed using the Wilson score method. Likelihood ratios were calculated as LR + = Sens/(1–Spec) and LR– = (1–Sens)/Spec, with diagnostic odds ratio DOR = LR+/LR–. All statistics were derived from the held-out test set and its confusion matrix; calibration and decision-curve analyses were performed on the held-out test set using the predicted probabilities exported by the model. Analyses were conducted in Python using pandas/NumPy and scikit-learn for modeling, XGBoost and LightGBM for boosting (16), and shap for model explainability (17). We used fixed random seeds (42) and split data at the participant level to support reproducibility. Key hyperparameters, software/library versions, and script details are provided in the Supplement (“Reproducibility Checklist”) in accordance with TRIPOD+AI transparency expectations (8).

### Model explainability

To aid interpretability, we applied SHAP (Shapley Additive Explanations) to obtain global feature importance and local case-level attributions (17). Representative plots illustrate why an individual is classified as high risk. We also consulted a recent review of explainable AI in healthcare to guide visualization and discussion of caveats (18).

## Results

### Multiple-model comparison and overall discrimination

**Figure 1:**
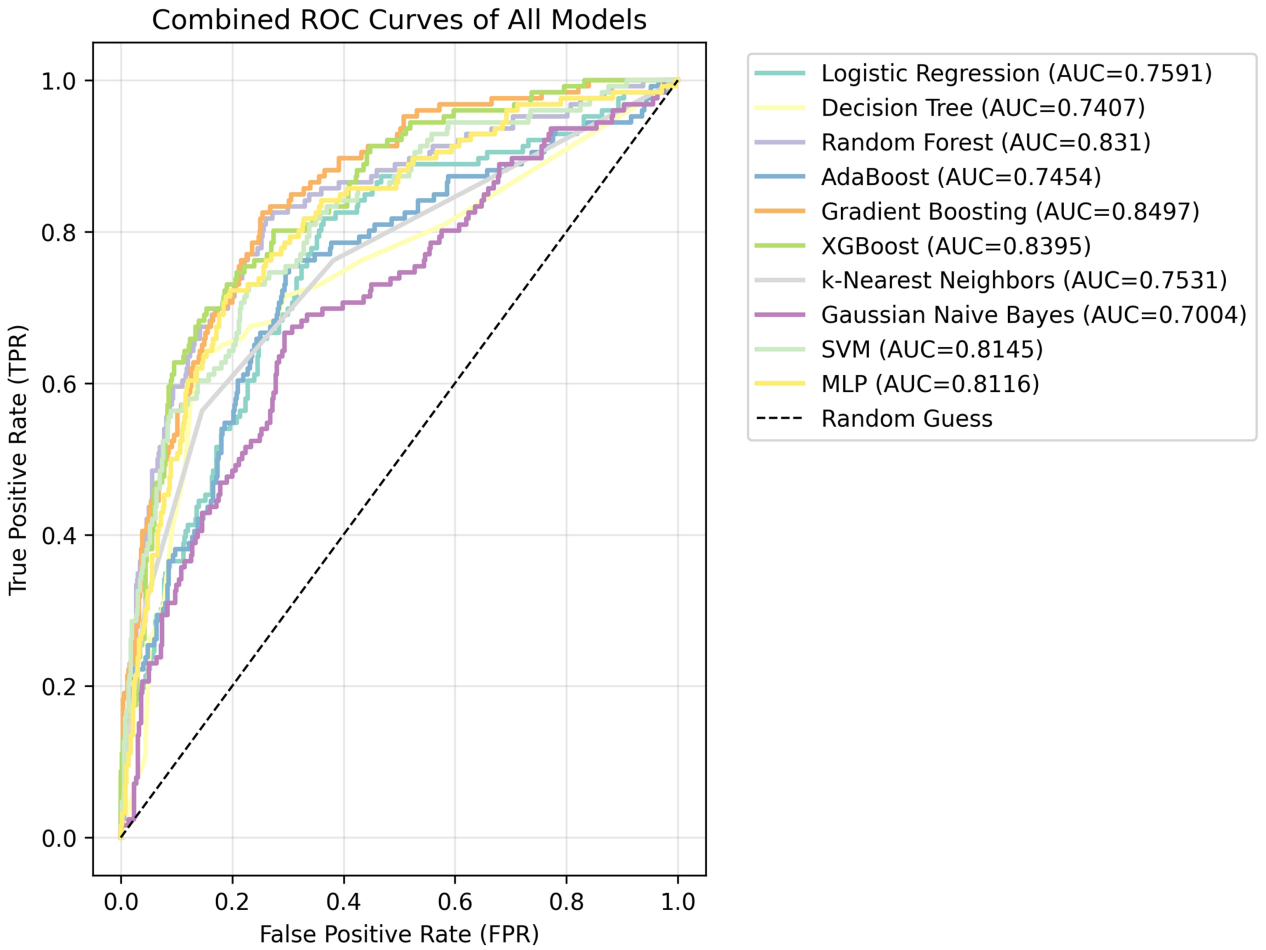
Combined ROC curves comparing ten algorithms.

We evaluated all 10 candidate algorithms on the held-out test set (Figure 1). Ensemble tree models performed best: Gradient Boosting and XGBoost achieved AUCs around 0.84–0.85, followed by Random Forest. SVM and logistic regression had intermediate performance, and Gaussian naïve Bayes had the lowest AUC. We present these results descriptively in Figure 1, without formal statistical comparisons.

### Cross-validation performance of Gradient Boosting and model selection

In 10-fold cross-validation, the Gradient Boosting model achieved mean accuracy 0.862 (SD 0.013), F1 score 0.371 (SD 0.052), and AUC 0.855 (SD 0.031). The relatively low F1 reflects the small fraction of positive cases, whereas the consistently high AUC and accuracy indicate consistent discrimination. Given its stable performance and high AUC, we selected Gradient Boosting as the primary model for testing (Table 1).

**Table 1:** 10-fold cross-validation performance (mean ± SD).

Metric	Mean ± SD
Accuracy	0.862 ± 0.013
F1 score	0.371 ± 0.052
AUC	0.855 ± 0.031

### Held-out test performance and operating characteristics

**Figure 2:**
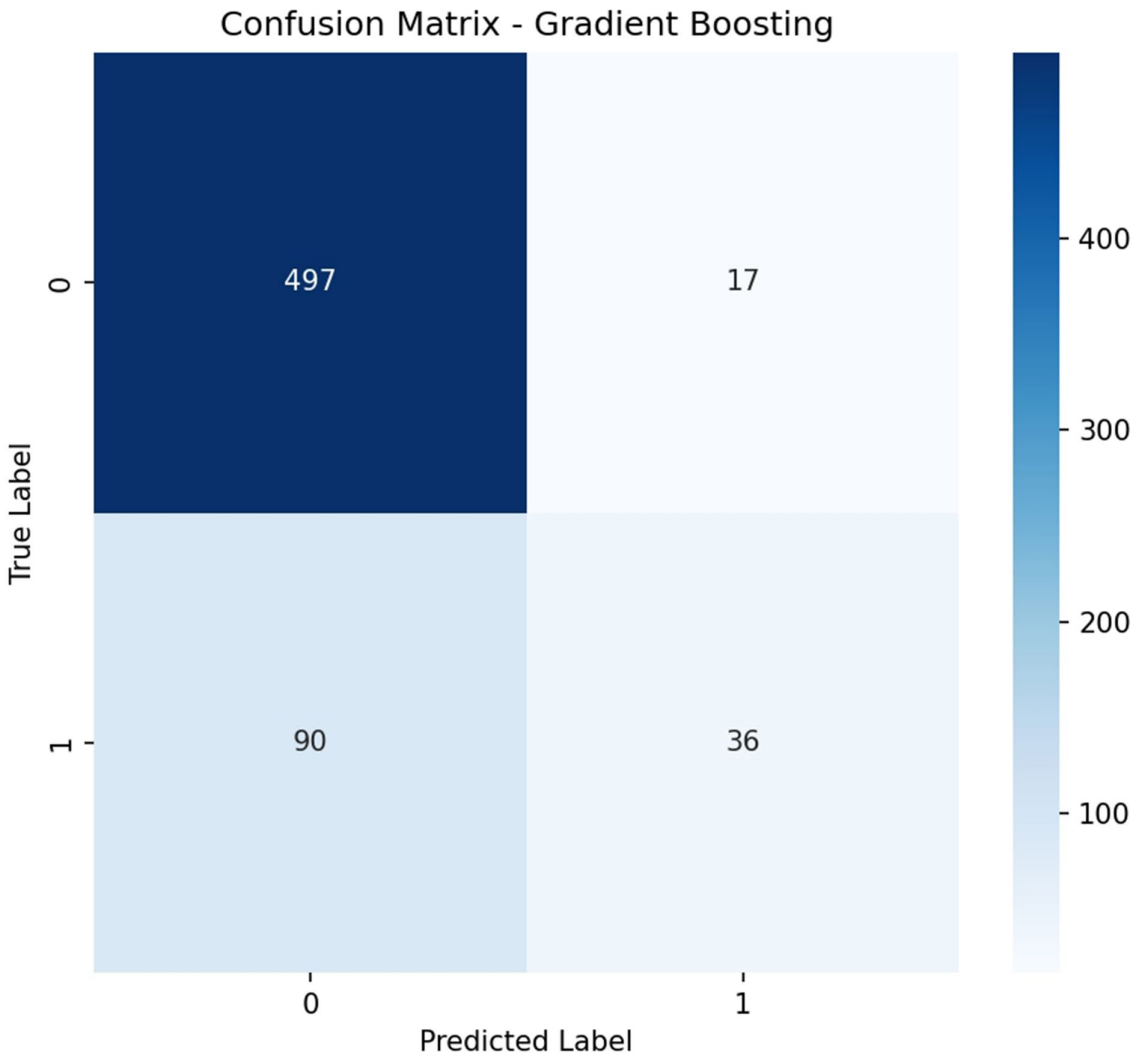
Confusion matrix for Gradient Boosting at the current operating threshold (TP = 36, FP = 17, FN = 90, TN = 497).

**Figure 3:**
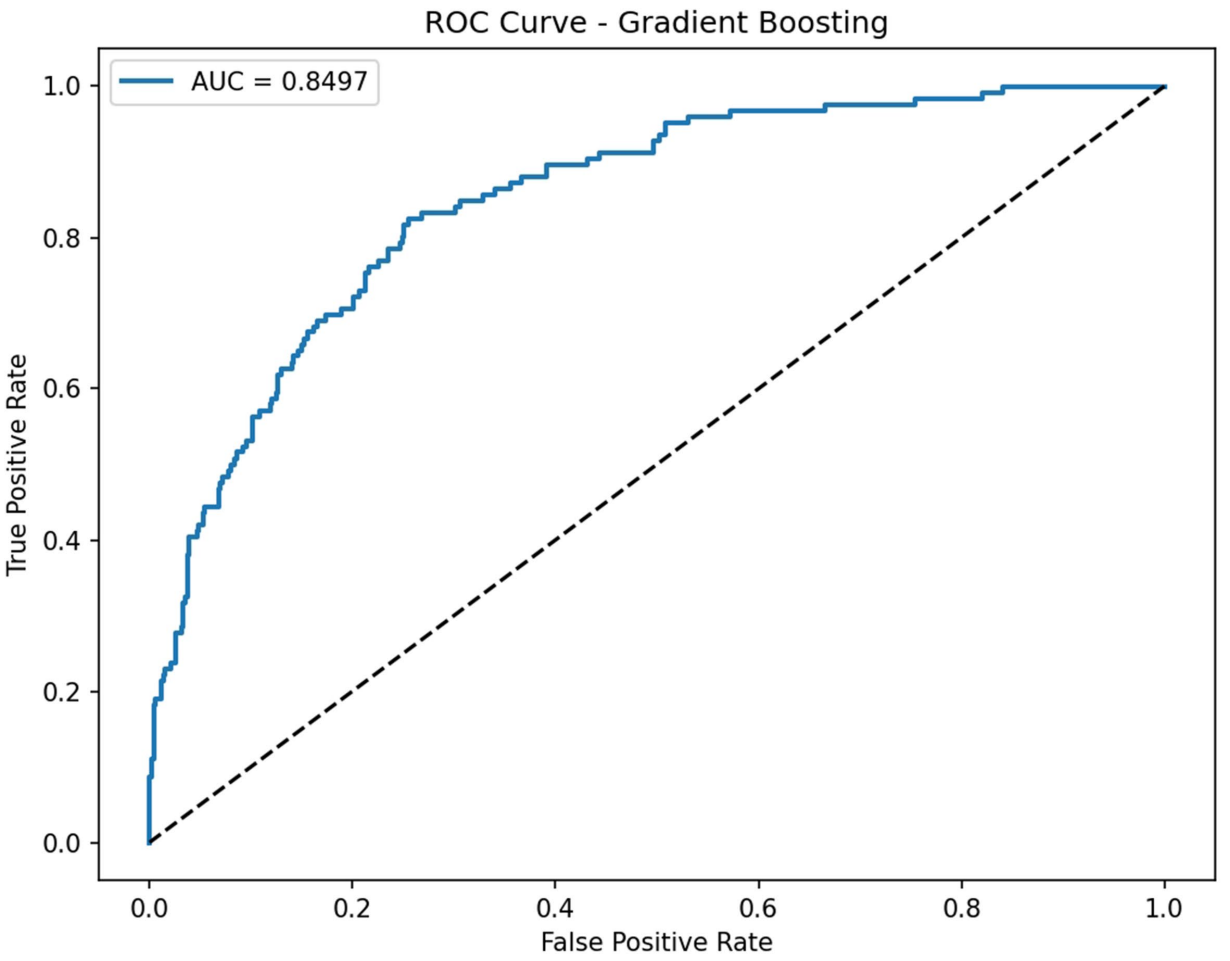
ROC for Gradient Boosting (AUC ≈ 0.850).

On the independent test set, the Gradient Boosting model attained AUC 0.850, accuracy 0.833, and F1 score 0.402 (Table 2). At the current operating threshold (0.50), the sensitivity was 0.286, specificity 0.967, PPV 0.679, and NPV 0.847, and the confusion matrix was TP = 36, FP = 17, FN = 90, TN = 497 (Figure 2), with the ROC curve is shown in (Figure 3). This operating point emphasizes high specificity at the cost of low sensitivity, as intended in initial evaluation. At a high-sensitivity threshold of 0.159, the model achieved a sensitivity of 0.802 with a specificity of 0.751 (PPV 0.441; NPV 0.939).

**Table 2:** Held-out test-set performance.

Metric	Test set
Accuracy	0.833
F1 score	0.402
AUC	0.850

### Model interpretability: global importance and individual attribution

**Figure 4:**
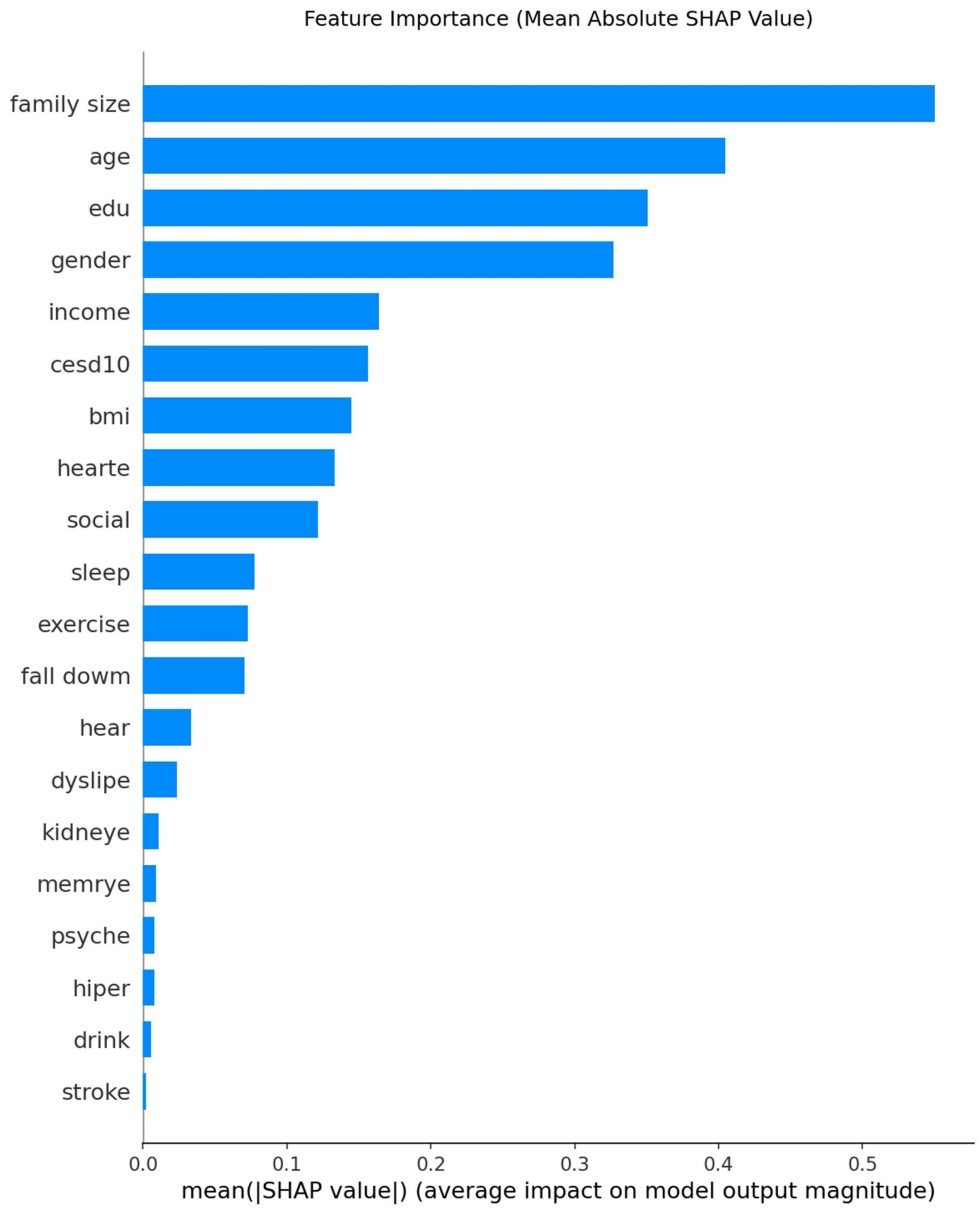
Global feature importance by mean absolute SHAP value.

**Figure 5:**
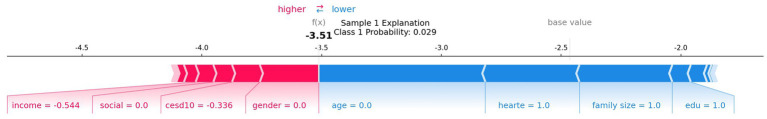
Representative SHAP force plot for one individual. AUC, area under the ROC curve; SHAP, Shapley Additive Explanations.

SHAP analysis identified family size, age, education, sex, income, and CES-D score as the most important predictors (Figure 4). At the individual level, a representative SHAP force plot (Figure 5) shows how protective factors (e.g., larger family size, higher education) can offset unfavorable factors (e.g., lower income, limited social participation), resulting in a lower predicted risk. We offer only descriptive interpretations and make no causal claims.

### Additional supplementary results

To conserve space, the 10-fold CV fold-wise variability (boxplots) and SHAP beeswarm/dependence plots (capturing non-linear patterns and interactions) are included in the Supplement (Supplementary Figures S1–S3); the main text cites only their key conclusions.

## Discussion

### Main findings and significance

Our Gradient Boosting model, trained on routine community outpatient data, achieved good discrimination between MCI and normal cognition (test set AUC ≈ 0.85). At the default threshold, the model had high specificity (~0.97) but low sensitivity (~0.29). Shifting to a high-sensitivity threshold (sensitivity ≈0.82) substantially increased false positives (specificity ≈0.64), reflecting the expected trade-off in screening contexts. These findings indicate that the model effectively identifies high-risk individuals, and that threshold adjustment can balance sensitivity versus specificity for clinical needs.

Our model’s performance is comparable to recent ML studies. For example, Fernández-Blázquez et al. built XGBoost models in older adults and reported AUC ≈ 0.836 (19). Similarly, Hu et al. found AUC ≈ 0.83 for cognitive impairment in a Chinese community cohort (20). These results suggest that readily available outpatient data can yield predictive accuracy on par with more complex approaches. Other studies further support this potential. Yan et al. reported that a simple logistic model in stroke patients achieved AUC 0.8595 (21), and a wearable-sensor study achieved near-perfect discrimination (AUC ≈ 0.94–1.0) by aggregating daily activity, sleep, and heart-rate signals (22). These findings show that both vascular risk profiles and digital physiological signals can provide strong predictive performance and may complement our approach. The top predictors in our model (age, female sex, education, social support, and depression) align with known risk factors (7, 20), supporting the model’s clinical plausibility. Together, our findings demonstrate that AI applied to routine clinical data may offer a practical, low-cost tool for early MCI screening in primary care, while leaving room for future integration of wearable or stroke-specific data.

Beyond discrimination and preset thresholds, we assessed model calibration and decision-curve (net benefit) analysis on the test set using individual predicted probabilities. These internal results suggest good calibration and positive net benefit across a range of thresholds. Nonetheless, external validation is needed to confirm the model’s usefulness. Nonetheless, the reliance on routinely collected EHR data and SHAP-based explanations underscores the feasibility of this approach in primary care settings.

### Comparison with previous studies

Few previous studies have focused on MCI prediction using non-imaging clinical data. Richter-Laskowska et al. used EMR features (demographics, labs, comorbidities) to distinguish MCI patients from healthy controls, achieving AUC ≈ 0.75 (23). Hu et al. applied ML to Chinese community data and obtained AUC ≈ 0.83 for cognitive impairment (MCI and dementia), outperforming a conventional screening test (20). In comparison, our model’s AUC (~0.85) was somewhat higher. This may reflect our inclusion of psychosocial factors (e.g., education, living situation) and consistent cross-validation, which can improve generalizability. Fernández-Blázquez et al. also built XGBoost models in older adults (including cognitive test scores) with AUC ≈ 0.836 (24). Yan et al. reported that a parsimonious logistic regression model in high-risk stroke patients achieved AUC 0.8595 (25). Xu et al. showed that aggregating physical activity, sleep, and heart-rate data from wearable trackers yielded AUC values around 0.94–1.0 for predicting MCI (26). Zhu et al. combined clinical variables with brain MRI markers (white-matter hyperintensity and baseline systolic blood pressure) in an XGBoost model, achieving AUC 0.9442 and accuracy 94.32% (27). These findings indicate that ML models using non-imaging predictors can reach AUCs in the range of ~0.75–0.85 and that incorporating vascular profiles, digital biomarkers, or imaging features may further improve performance. Overall, our results are broadly consistent with the literature and suggest that ML on routine clinical features could enhance community cognitive screening while leaving room for augmentation with more advanced data modalities.

### Mechanisms and interpretability

The model’s top features have clear links to known mechanisms of cognitive decline. Older age and female sex increased predicted MCI risk in our SHAP analysis, consistent with established epidemiology. Education and social engagement appeared as protective factors: the 2020 Lancet Commission highlighted education, physical activity, and social contact as key factors that reduce dementia risk (28). Chronic conditions (hypertension and diabetes) act via vascular and metabolic pathways, as illustrated by the stroke-risk model in which transient ischemic attack, diabetes, and hypertension were the top predictors (21). Frequent outpatient visits or high chronic disease counts likely reflect overall health burden and were associated with higher predicted risk. Depressive symptoms (high CES-D scores) also emerged as important, aligning with evidence that depression is a modifiable risk factor for dementia (29).

Digital biomarker studies further underscore relevant pathways. For example, Xu et al. found that heart-rate variability and physical activity features were among the most important predictors of MCI (30), linking autonomic and activity factors to cognitive decline. Imaging-based models offer complementary insights: Zhu et al. reported that white-matter hyperintensity scores and elevated systolic blood pressure were top features in their XGBoost model (27), supporting vascular contributions to MCI. In summary, our SHAP explanations show that age, sex, education, social support, and mood drive the model’s predictions in a clinically plausible manner. This concordance with known clinical and emerging factors increases confidence in the model and may help clinicians understand and trust its predictions in practice.

### Threshold trade-offs and screening context

The choice of operating threshold has important practical implications. In our study, raising the threshold to achieve ~80% sensitivity markedly increased the false-positive rate. In community screening, higher sensitivity (at the cost of lower specificity) may be preferred to capture most true cases, whereas in specialized or resource-limited settings a higher threshold could reduce unnecessary referrals. This trade-off must be carefully considered: many false positives would lead to extra referrals, testing, and patient anxiety, while the default high-specificity setting would miss many true cases. Ultimately, the optimal threshold depends on local resources and acceptable risk levels. Using the individual predicted probabilities, we derived operating thresholds and evaluated net benefit via decision-curve analysis to identify clinically relevant operating points (31).

### Calibration and clinical utility

**Figure 6:**
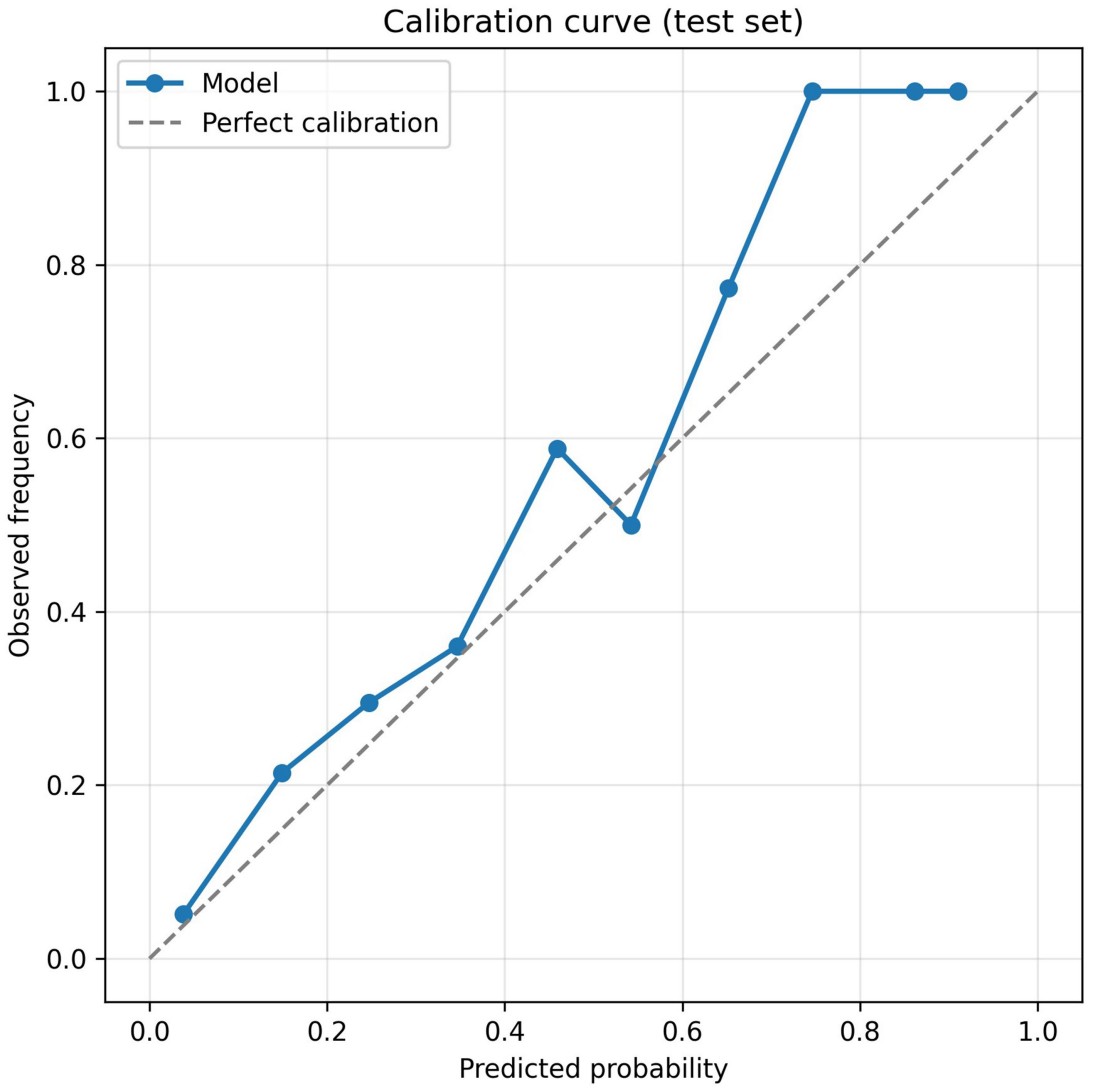
Calibration curve for the Gradient Boosting model on the test set.

**Figure 7:**
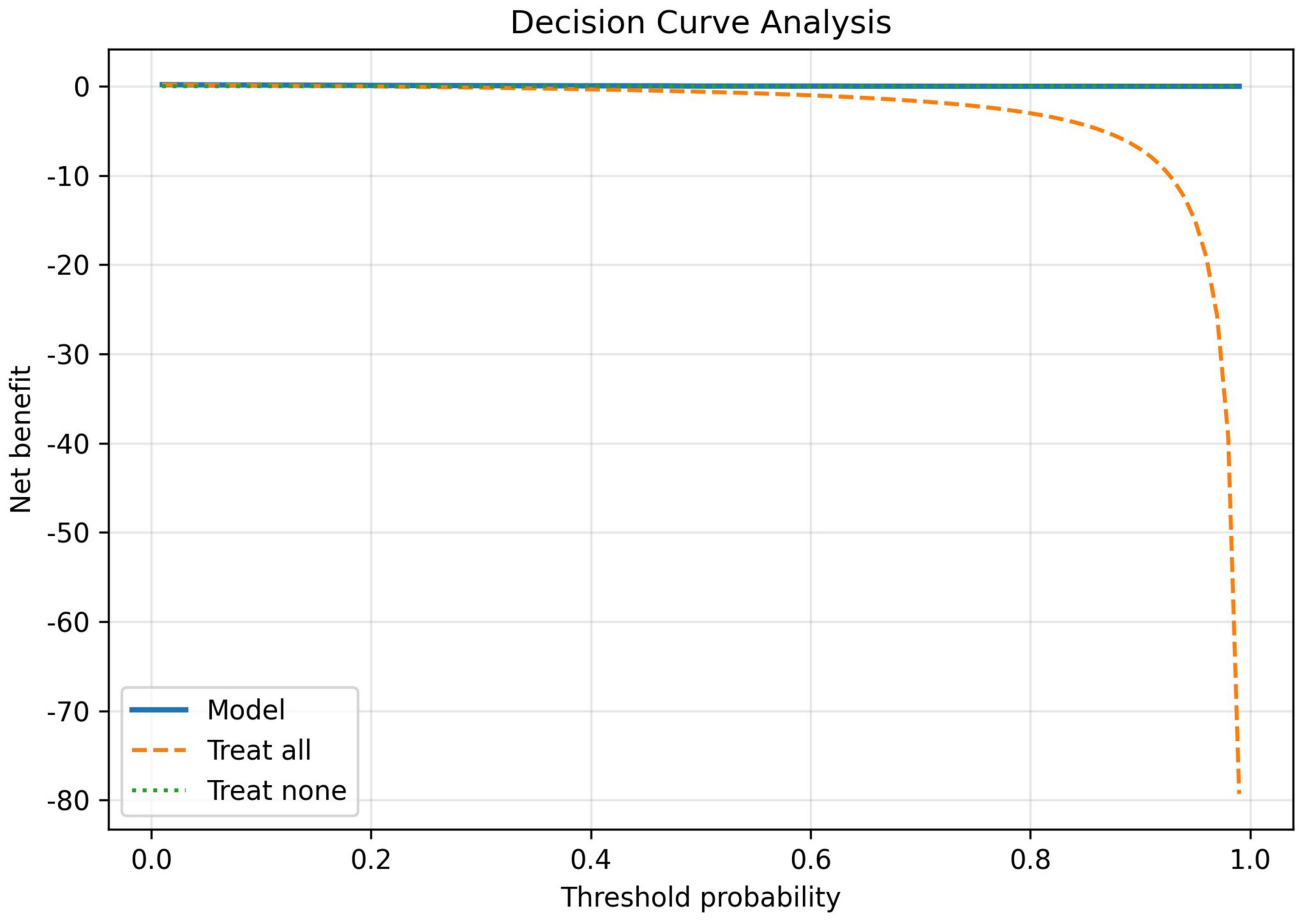
Decision-curve analysis for the Gradient Boosting model (net benefit vs. threshold probability for the model, “screen-all,” and “screen-none” strategies).

Beyond discrimination, a valid prediction model should be well calibrated and its clinical net benefit evaluated. The calibration curve (Figure 6) showed close agreement between predicted probabilities and the observed incidence of MCI, indicating good calibration. As shown in Figure 7, the decision-curve analysis demonstrated that the Gradient Boosting model provided a higher net benefit than both the “screen-all” and “screen-none” strategies across most threshold probabilities, suggesting potential clinical utility.

### External validity and bias assessment

We acknowledge several limitations and potential sources of bias. First, our sample is from community health centers in one region, so selection bias is possible and generalizability is uncertain. Second, predictors were extracted from routine EHR data, which may contain misclassification or missing entries. Third, the MCI outcome was based on outpatient cognitive screening (e.g., MoCA or AD8) rather than biomarker confirmation, introducing potential misdiagnosis. Finally, we have only performed internal validation; no external cohort was tested. These factors (participant selection, predictor/outcome measurement, and analysis) could introduce bias or limit performance elsewhere. For example, a cross-sectional study in northern Peru found high prevalence of MCI and dementia and identified age, low education, hypertension, and hearing loss as significant factors (32). Such differences in prevalence and risk profiles suggest that models developed in one region may not perform as well elsewhere. We attempted to mitigate overfitting through cross-validation, but future work must test the model in independent cohorts to confirm its transportability and adjust for any bias, using tools such as the Prediction model Risk Of Bias Assessment Tool (PROBAST) (12).

We also recognize the importance of monitoring model fairness across demographic and socioeconomic subgroups and of adapting to potential distribution shifts over time. Plans for periodic recalibration, updating, and performance auditing will therefore be included in our external validation protocol.

### Application prospects and future work

Our results suggest potential for developing a decision-support tool to aid community dementia screening; however, such a tool should only be pursued after external validation, calibration, and net-benefit analyses confirm its clinical value. Inspired by similar work [e.g., Fernández-Blázquez et al. provided an online MCI risk calculator using non-imaging data (7)], we plan to package our model into a user-friendly application contingent on further validation. Future tools might also incorporate variables identified in high-risk stroke cohorts (e.g., transient ischemic attack, diabetes, education, hypertension) (21) and include digital biomarkers. The wearable-sensor study shows that aggregating physical activity, sleep, and heart-rate signals yields high discrimination (22), suggesting that mobile devices and telehealth could enrich risk stratification. Before real-world deployment, further steps are needed: prospective validation in external cohorts, integration with EHR systems, and evaluation of usability and cost-effectiveness. Pilot studies could assess how using the model affects referral rates and early diagnosis in practice. In the long term, such tools could enable more targeted cognitive assessments and preventive interventions for at-risk older adults.

## Conclusion

In summary, machine learning applied to routine community outpatient data can identify older adults at risk of mild cognitive impairment. Our Gradient Boosting model provided good discrimination (AUC ≈ 0.85) and highlighted well-known risk factors (age, education, depression, etc.) in its predictions. Although further validation is needed, this approach could augment existing screening programs. By enabling earlier detection of at-risk individuals, a deployed model could prompt timely cognitive assessments and interventions to slow cognitive decline.

## Data Availability

The original contributions presented in the study are included in the article/Supplementary material, further inquiries can be directed to the corresponding author.
