# Supplementary material for: AI-assisted screening for mild cognitive impairment using routine EHR data: a Gradient Boosting approach
Source: Front Neurol. 2026 Feb 17;17:1718791. doi: 10.3389/fneur.2026.1718791 (PMC12954454; doi:10.3389/fneur.2026.1718791)
Supplement: Supplementary file 1 [file Data_Sheet_1.ZIP › Supplementary.docx]

**Appendices and Availability Statements**

**Appendix A. Predictor definitions and preprocessing**

This study used routinely collected primary care EHR data from outpatients aged 60 years or older in Zhejiang China. MCI was adjudicated according to the 2018 Chinese guideline. Predictors were derived from routine records including demographics education comorbidities medications vital signs laboratory results and health care utilization.

Categorical variables were encoded. Continuous variables were scaled. Missing values were imputed using procedures fit on the training split only. All preprocessing steps were fit on the training data and then applied to the held out test set. Variable definitions and transformation rules were prespecified and applied consistently.

Data were split at the patient level into a development set and an independent test set with stratification by outcome prevalence. Random seeds were fixed to support reproducibility.

**Appendix B. Model development and internal validation details**

We developed several supervised learning models using the training data and selected the Gradient Boosting model based on discrimination on cross validation. Internal validation used stratified 10 fold cross validation on the training data. Performance on the independent test set was reported once for the final model.

Discrimination was measured by AUC. Overall performance was measured by accuracy and F1. At the current operating threshold sensitivity specificity PPV and NPV were computed from the test set confusion matrix. Predicted probabilities were generated and used to perform calibration and decision‑curve analyses, and these results are reported in the revised manuscript.

The current operating point emphasizes high specificity with lower sensitivity. Alternative high sensitivity thresholds will be evaluated in external validation.

**Appendix C. Software and reproducibility checklist**

Analyses were conducted in Python. A list of required packages is provided in the file requirements.txt; exact versions are available upon request. The analysis notebook is mci-ml-pipeline-modified.ipynb. Documentation is provided in README.md. These files are bundled in Supplementary Code.zip. Random seeds and patient level splits were fixed. All preprocessing was fit only on the training folds or split. The test set remained untouched until final evaluation.

**Appendix D. Reporting checklists TRIPOD AI and PROBAST brief**

We completed the TRIPOD AI and PROBAST checklists to support transparent reporting and appraisal of risk of bias and applicability.

Study type and setting. Retrospective model development with internal validation in primary care. External validation is planned.

Participants predictors and outcome. Eligibility criteria predictor definitions and handling and MCI adjudication are specified in Methods and Appendix A.

Sample size and missing data. Sample size rationale and missing data handling are reported in Methods. Imputation and transformations were confined to the training data.

Analysis. Model specification resampling strategy and performance metrics are detailed in Methods and Appendix B.

Both will be evaluated in future work.

**Data Availability Statement**

The dataset cannot be shared publicly due to privacy restrictions. Controlled access can be considered on reasonable request to the corresponding author and subject to institutional approvals.

**Code Availability Statement**

All analysis code is provided as Supplementary Code.zip including mci-ml-pipeline-modified.ipynb.README.md and requirements.txt. If the submission system does not support code uploads the code will be deposited in an open repository and the DOI supplied at revision or upon request.
